# Age- and Sex-Dependent Association between *FTO* rs9939609 and Obesity-Related Traits in Chinese Children and Adolescents

**DOI:** 10.1371/journal.pone.0097545

**Published:** 2014-05-14

**Authors:** Meixian Zhang, Xiaoyuan Zhao, Hong Cheng, Liang Wang, Bo Xi, Yue Shen, Dongqing Hou, Jie Mi

**Affiliations:** 1 Department of Epidemiology, Capital Institute of Pediatrics, Beijing, China; 2 Graduate School, Peking Union Medical College, Beijing, China; 3 Department of Biostatistics and Epidemiology, College of Public Health, East Tennessee State University, Johnson City, Tennessee, United States of America; 4 Department of Maternal and Child Health Care, School of Public Health, Shandong University, Jinan, China; Tulane School of Public Health and Tropical Medicine, United States of America

## Abstract

**Background:**

The associations between common variants in the fat mass- and obesity-associated (*FTO*) gene and obesity-related traits may be age-dependent and may differ by sex. The present study aimed to assess the association of *FTO* rs9939609 with body mass index (BMI) and the risk of obesity from childhood to adolescence, and to determine the age at which the association becomes evident.

**Methods:**

Totally 757 obese and 2,746 non-obese Chinese children aged 6–18 years were genotyped for *FTO* rs9939609. Of these, a young sub-cohort (*n* = 777) aged 6–11 years was reexamined 6 years later. Obesity was defined using the sex- and age-specific BMI cut-offs recommended by the International Obesity Task Force.

**Results:**

The associations of *FTO* rs9939609 with BMI and obesity did not appear until children reached 12–14 years. The variant was associated with an increased BMI in boys (*β = *1.50, *P = *0.004) and girls (*β = *0.97, *P = *0.018), respectively. Thereafter, the magnitude of association increased in girls at ages 15–18 years (*β = *2.02, *P*<0.001), but not boys (*β = *0.10, *P*>0.05). Age was found to interact with the variant on BMI (*P*<0.001) and obesity (*P* = 0.042) only in girls. In the sub-cohort, the associations of *FTO* rs9939609 with BMI (*β = *1.07, *P = *0.008) and obesity (OR = 2.09, 95% CI: 1.12, 3.91) were only observed 6 years later (ages 12–18 years) in girls, even after adjusting for baseline BMI.

**Conclusions:**

The association between *FTO* rs9939609 and obesity-related traits may change from childhood to adolescence in Chinese individuals, and the association may start as early as age 12 years, especially in girls.

## Introduction

The increasing prevalence of childhood obesity has had a massive impact on global health [1]. Genetics has a remarkable effect on obesity in an obesogenic environment [2]. A systematic review of genetic studies concluded that the genetic contribution to body mass index (BMI) may increase from childhood into adolescence, and it may have a greater influence during childhood than adulthood [3]. The fat mass- and obesity-associated gene (*FTO*) was the first, well-replicated gene to be associated with common obesity in both adults and children from various ethnic backgrounds [4], [5]. A twin study in a longitudinal sample showed that the magnitude of the effect of a common variant in the *FTO* gene on BMI increased in parallel with a rise in heritability with increasing age during childhood [6]. Evidence from several studies indicates that the association between *FTO* variants and obesity-related traits may be age-dependent and may differ by sex [7]–[10]. However, the age-related patterns of associations have differed among studies. The association has been variably reported to begin during the neonatal period [11], childhood [7], and adolescence [12]. All of these studies were performed on individuals of European ancestry. Recently, a replication study in Shanghai children aged 10–12 years confirmed associations of a single-nucleotide polymorphism (SNP) rs9939609 in *FTO* intron 1 with obesity indices and that this association differs in males and females [10]. Our previous study also confirmed overall associations of *FTO* rs9939609 with BMI and the risk of obesity in Chinese children aged 6–18 years, but this study did not find any associations of the variant with birth weight [13]. The pattern and age of onset of the association between *FTO* rs9939609 and BMI/obesity from childhood to adolescence in a Chinese population are not clear.

To our knowledge, no prospective studies have examined the age- and sex-specific associations of genetic variants with obesity-related traits in Chinese children. In the present study, we tracked the associations between *FTO* rs9939609 and obesity-related traits from childhood into adolescence, and examined interactions of this variant with age and sex on obesity-related traits.

## Subjects and Methods

### Study Design and Subjects

As part of the population-based cross-sectional Beijing Child and Adolescent Metabolic Syndrome (BCAMS) study [14], more than 3,500 unrelated Han Chinese children aged 6–18 years were recruited for venipuncture blood samples in 2004. The present study includes 757 obese (549 boys, 72.5%) and 2,746 non-obese children (1,232 boys, 44.9%) based on the age- and sex-specific BMI cut points recommended by the International Obesity Task Force (IOTF) [15]. To obtain the high follow-up rate, we only focused on the young sub-cohort of 1,620 children between 6 to 11 years at baseline. However, only 777 subjects (48.0%) including 246 obese (58.6% of those eligible) and 531 non-obese children (44.3% of those eligible) were reassessed for BMI in December 2010. The study was approved by the Ethics Committees Review Board of Capital Institute of Pediatrics, Beijing, China. Written informed consent was obtained from all children and from their parents or guardians.

### Anthropometric Measurements and Questionnaire

Anthropometric measurements included weight, height, waist circumference, and fat mass percentage by bioelectrical impedance analysis (TANITA TBF-300A). Weight was measured to the nearest 0.1 kg on a balance-beam scale, and height was measured to the nearest 0.1 cm with a wall-mounted stadiometer. Pubertal developmental stage was determined by trained physicians, according to the criteria described by Marshall and Tanner [16]. Puberty was defined as breast development of tanner stage II or later for girls and testicular volume of 4 ml or more for boys. Lifestyle habits (exercise and eating habit) were measured by a validated questionnaire [17]. Sedentary behavior was determined by the time spent either watching television or playing video/computer games per day in a week. The intensity of physical activity was estimated by the total days per week in which subjects spent at least 30 minutes per day participating in extracurricular physical activities. Frequency of meat consumption was assessed and categorized into every day, 3–5 times per week, and less than 2 times per week.

### Genotyping

Genomic DNA was isolated from peripheral white blood cells using the salt fractionation method. The details of the genotyping methods for rs9939609 have been described elsewhere [13]. In short, the rs9939609 SNP in the *FTO* gene was genotyped by allele-specific real-time polymerase chain reaction (RT-PCR) using an ABI 5700 Real Time PCR Instrument (Applied Biosystems, Foster City, CA, U.S.) [18]. DNA samples were genotyped for a single SNP using an equal aliquot of sample in two allele-specific PCR reactions. The genotype was determined from the cycle threshold (Ct) values obtained with the GeneAmp 5700 SDS software. The genotyping call rate was 95.73% for the cohort after the first genotyping reaction, and 99.97% of the samples’ genotypes were found by re-genotyping. The estimated genotyping error rate was found less than 1% by validating 100 random samples of known genotype in additional reactions.

### Statistical Analysis

Hardy-Weinberg equilibrium was performed using Pearson’s chi-squared test. Assuming an additive mode of inheritance, multivariable linear regression models were used to test for an association between obese indices and the SNP genotype. Multiple logistic regression models were performed to estimate the association between the variant and obesity. The interaction between age and *FTO* genotype was evaluated using interaction terms in the regression models. Statistical analyses were performed using the Statistical software SPSS, version 20.0 (IBM Corp. Released 2011. IBM SPSS Statistics for Windows, Version 20.0. Armonk, NY: IBM Corp.). Two-sided *P* values <0.05 were considered statistically significant.

## Results

### Baseline Characteristics of Participants

Subjects ranged from 6 to 18 years old (mean age 12.4±3.1 years) and distributed across all Tanner stages. Girls had a more advanced pubertal development than boys (80.2% vs. 61.0%, *P*<0.001). Of the 3,503 study subjects, the frequency of the A allele was 12.1%. The SNP rs9939609 genotype frequencies in each subgroup met Hardy-Weinberg expectations (all *P*>0.05, data not shown).

Characteristics of participants at baseline according to weight status are summarized in Table 1. In the total cohort, obese children were more likely to be male, younger, risk A allelic distribution, and to engage in more sedentary behavior than non-obese children. In the sub-cohort, significant differences in sex and pubertal development were observed between the obese and non-obese children.

**Table 1:** Baseline Characteristics of Study Participants in the Beijing Child and Adolescent Metabolic Syndrome Study, 2004, China^a^.

	Total cohort (*n* = 3,503)			Sub-cohort (*n* = 777)		
	Obese^b^ (*n* = 757)	Non-obese (*n* = 2,746)	*P-* value	Obese^b^ (*n* = 246)	Non-obese (*n* = 531)	*P-* value
Boys	72.5	44.9	<0.001	69.9	46.7	<0.001
Age, years			<0.001			0.520
6–8	20.2	14.7		38.2	40.7	
9–11	35.3	29.0		61.8	59.3	
12–14	27.9	30.9		0	0	
15–18	16.6	25.4		0	0	
Puberty^c^	60.4	73.3	<0.001	27.1	34.5	0.044
Sedentary behavior (≥2 hours/day)	51.3	45.5	0.006	52.5	48.7	0.338
Physical activity level^d^			0.103			0.101
High	47.1	42.8		37.6	35.4	
Moderate	33.9	36.1		33.5	41.1	
Low	18.9	21.1		28.9	23.5	
Frequency of meat consumption			0.695			0.576
Everyday	42.9	44.6		41.2	38.8	
3–5 times/week	20.0	19.8		23.5	21.9	
≤2 times/week	37.1	35.6		35.4	39.3	
*FTO* rs9939609			0.032			0.290
TT	74.9	78.3		73.2	77.8	
TA	22.5	20.2		24.0	20.5	
AA	2.6	1.5		2.8	1.7	

Abbreviation: *FTO*, fat mass- and obesity-associated gene.

a Data are expressed as frequency and differences between obese and non-obese groups were examined using the Chi-square test.

b Obesity was defined using the age- and sex-specific BMI cutoff points recommended by the International Obesity Task Force (IOTF).

c Puberty was defined as breast development of tanner stage II or later for girls and testicular volume of 4 ml or more for boys.

d Physical activity level was defined as ≥30 minutes per day with the following frequencies: high, ≥5 days/week; moderate, 3–4 days/week; and low, <3 days/week.

### Cross-sectional Results

#### Age-specific associations between FTO and BMI in boys and girls

The subjects were grouped into four age categories: 6–8 years (*n* = 557), 9–11 years (*n* = 1,063), 12–14 years (*n* = 1,059), and 15–18 years (*n* = 824). Table 2 shows the associations between rs9939609 and BMI by sex and age group. Statistically significant associations of rs9939609 with BMI did not appear until children reached 12–14 years of age after adjusting for sex and age. In addition, the magnitude of the association between the variant and BMI increased at 15–18 years of age only among girls (*β = *2.02; 95% CI: 1.13, 2.91). Age had an interaction with the variant on BMI only in girls (*P*<0.001 for *FTO* by age interaction). These associations remained significant even after adjusting for pubertal stage, sedentary behavior, physical activity, and meat consumption.

**Table 2:** BMI Profiles of Participants with Different *FTO* rs9939609 Genotypes and Cross-sectional Associations between *FTO* and BMI by Sex and Age.

Sex	Age (years)	*n*	BMI, kg/m^2^, Mean (SD)			*P* for Trend	Difference in BMI per A allele^a^	
		(TT/TA/AA)	TT	TA	AA		*β*	95%CI
All	6–8	440/109/8	18.7 (4.0)	18.7 (4.3)	19.9 (3.5)	0.79	0.1	−0.61, 0.81
	9–11	807/235/21	21.1 (4.4)	21.7 (4.3)	21.5 (4.7)	0.078	0.48	−0.05, 1.01
	12–14	826/215/18	22.4 (5.0)	23.6 (4.7)	24.7 (6.0)	**<0.001**	1.2	0.57, 1.84
	15–18	645/165/14	23.7 (4.9)	24.8 (4.6)	25.3 (4.9)	**0.002**	1.07	0.39, 1.75
	All	2718/724/61	21.7 (4.9)	22.5 (4.9)	23.0 (5.3)	**<0.001**	0.79	0.47, 1.10
Boys	6–8	227/65/6	19.2 (4.2)	19.9 (4.5)	20.7 (3.8)	0.153	0.74	−0.27, 1.75
	9–11	445/123/12	21.9 (4.6)	22.2 (4.5)	23.8 (3.5)	0.167	0.54	−0.23, 1.31
	12–14	400/105/6	23.3 (5.3)	24.1 (4.7)	31.2 (3.2)	**0.004**	1.5	0.49, 2.51
	15–18	308/75/9	25.5 (5.1)	25.8 (4.7)	24.9 (4.3)	0.847	0.1	−0.93, 1.14
	All	1380/368/33	22.6 (5.2)	23.2 (5.0)	24.9 (4.9)	**0.003**	0.71	0.24, 1.18
Girls	6–8	213/44/2	18.1 (3.6)	16.9 (3.1)	17.5 (1.2)	0.063	−1	−2.05, 0.06
	9–11	362/112/9	20.2 (4.1)	21.1 (3.9)	18.4 (4.5)	0.299	0.4	−0.35, 1.15
	12–14	426/110/12	21.6 (4.6)	23.1 (4.6)	21.4 (4.1)	**0.018**	0.97	0.17, 1.77
	15–18	337/90/5	22.0 (4.4)	24.1 (4.4)	26.1 (6.3)	**<0.001**	2.02	1.13, 2.91
	All	1338/356/28	20.8 (4.4)	21.9 (4.7)	21.0 (5.2)	**<0.001**	0.88	0.45, 1.30

Abbreviations: BMI, body mass index; CI, confidence interval; *FTO*, fat mass- and obesity-associated gene.

a Linear regression model was used to estimate the difference in BMI per A allele and 95% CI adjusted for sex and age.

Associations between the SNP and phenotypes waist circumference, waist-to-height ratio, and fat mass percentage were also evaluated. Like BMI, statistically significant associations were observed among adolescents aged 12–14 years onward, and more pronounced in girls (Table S1–S3).

#### Age-specific associations between FTO and obesity in boys and girls

The frequency of A allele of *FTO* rs9939609 was higher in obese children than in the non-obese (13.9% vs 11.6%; *χ^2^* = 6.894; *P = *0.032). The *FTO* rs9939609 A-allele was associated with an increased risk of obesity, with a per-allele OR of 1.23 (95% CI: 1.04, 1.46; *P* = 0.017) after adjusting for sex and age. The association differed across age groups, becoming significant at ages 12–14 years (OR = 1.46; 95% CI: 1.07, 2.01; *P* = 0.018). Both in boys and girls, the ORs increased across four age categories, although they did not reach statistical significance (data not shown). The interaction between genotype and age on risk of obesity was significant in girls (*P* = 0.042) but not in boys (*P*>0.05).

Age group was then replaced with pubertal stage. The associations of *FTO* with BMI and risk of obesity stratified by sex and pubertal stage are summarized in Table 3. Statistically significant associations were observed only after the onset of puberty and were more pronounced in girls as compared to boys.

**Table 3:** Cross-sectional Associations of *FTO* rs9939609 with Obesity Stratified by Sex and Pubertal Stage.

Sex	Pubertal stage	*OR*	95%CI	*P* value^a^
All	Prepuberty	1.11	0.81, 1.52	0.504
	Puberty^b^	1.36	1.09, 1.69	**0.007**
Boys	Prepuberty	1.28	0.90, 1.81	0.168
	Puberty^b^	1.25	0.94, 1.68	0.127
Girls	Prepuberty	0.65	0.28, 1.49	0.305
	Puberty^b^	1.48	1.06, 2.06	**0.020**

Abbreviations: CI, confidence interval; *FTO*, fat mass- and obesity-associated gene; OR, odds ratio.

a Adjusted for sex, age, sedentary behavior, physical activity, and meat consumption.

b Puberty was defined as breast development of tanner stage II or later for girls and testicular volume of 4 mL or greater for boys.

### Longitudinal Results

#### Change in association during the 6-year follow-up

In the sub-cohort of 777 children, the association of *FTO* with BMI and obesity was examined at baseline and during follow-up. As shown in Table 4, no statistically significant associations of *FTO* and childhood BMI and obesity were observed at baseline, but the associations during adolescence were significant 6 years later (ages 12–18 years) in girls, even after adjusting for baseline BMI. The association remained statistically significant after further adjusting for sedentary behavior, physical activity, and meat consumption at baseline.

**Table 4:** Sex-specific Associations of *FTO* rs9939609 with BMI a and Obesity b from Childhood into Adolescence among the Sub-cohort of 777 Children.

Sex	Phenotype Trait	At baseline (6–11 years)					At 6-year follow-up (12–18 years)				
		TT	TA	AA	Effect per A allele^c^	*P* value	TT	TA	AA	Effect per A allele^d^	*P* value
All (*n* = 777)	BMI, kg/m^2^	20.4 (4.3)	21.2 (4.6)	21.4 (4.2)	0.48 (0.30)	0.113	24.9 (5.4)	26.5 (5.6)	26.7 (5.0)	0.76 (0.25)	**0.003**
	Obesity	1.00	1.22 (0.84, 1.77)	1.67 (0.59, 4.66)	1.24 (0.91, 1.70)	0.176	1.00	1.64 (1.12, 2.38)	2.16 (0.77, 6.05)	1.56 (1.05, 2.33)	**0.029**
											
Boys (*n* = 420)	BMI, kg/m^2^	21.2 (4.5)	21.9 (4.7)	22.4 (3.8)	0.59 (0.42)	0.16	25.8 (5.5)	27.2 (5.6)	26.9 (4.5)	0.50 (0.32)	0.122
	Obesity	1.00	1.19 (0.75, 1.90)	2.28 (0.63, 8.26)	1.29 (0.88, 1.90)	0.196	1.00	1.52 (0.95, 2.44)	1.32 (0.36, 4.84)	1.25 (0.74, 2.11)	0.411
											
Girls (*n* = 357)	BMI, kg/m^2^	19.5 (3.9)	20.4 (4.3)	19.8 (4.7)	0.38 (0.44)	0.394	24.0 (5.1)	25.7 (5.4)	26.3 (6.1)	1.07 (0.40)	**0.008**
	Obesity	1.00	1.28 (0.68, 2.40)	0.80 (0.09, 6.97)	1.16 (0.67, 2.00)	0.592	1.00	1.80 (0.97, 3.34)	4.86 (0.95, 24.86)	2.09 (1.12, 3.91)	**0.021**

Abbreviations: BMI, body mass index; *FTO*, fat mass- and obesity-associated gene.

a BMI was calculated as weight in kilograms divided by height in meters squared, and expressed as mean (SD).

b Obesity was diagnosed using the age- and sex-specific BMI cutoff points recommended by the International Obesity Task Force (IOTF), and expressed as odds ratio (OR) and 95% confidence interval (CI) under a genotypic model.

c Adjusted for sex and age at baseline.

d Adjusted for sex, age at follow up, and BMI at baseline.

#### Influence of FTO on incidence of obesity and increase in BMI during follow-up

At the 6-year follow-up, the incidence of obesity was 10.9% (58/531), and 64.2% (158/246) of obese children were still obese from childhood to adolescence. When stratified by obese status at baseline, a significantly higher risk of incident obesity was observed for children carrying the rs9939609 A allele (OR = 2.64; 95% CI: l.52, 4.57; *P*<0.001) versus those carrying the T allele after adjusting for sex, age at follow-up, and BMI at baseline. However, the variant was not associated with maintaining obesity (OR = 0.96; 95% CI: 0.56, 1.64; *P* = 0.881).

The association of the variant with changes in BMI during 6-year follow-up was also evaluated. The present results showed a BMI increase was associated with the more copies of the A allele the subject had (*β* = 0.77 kg/m^2^, 95% CI: 0.28, 1.27; *P* = 0.002). The increase in BMI was more pronounced in girls (*β = *1.07 kg/m^2^, 95% CI: 0.28, 1.87; *P* = 0.008) than in boys (*β = *0.53 kg/m^2^, 95% CI: −0.10, 1.16; *P* = 0.099).

## Discussion

This is the first study to examine age-related association between *FTO* rs9939609 and obesity-related traits in Chinese children. We found positive associations between the common variant rs9939609 and BMI and the risk of obesity. In addition, the associations changed across age groups from childhood to adolescence, with beginning to be significant at 12–14 years and persisting into late puberty in girls but not in boys.

Various studies have reported that *FTO* is a susceptibility gene underlying polygenic obesity. Several studies have shown that the influence of *FTO* on BMI changes over the life span in European populations [19], [20]. However, the findings of the age of onset of the association were mixed. In the current study, the cross-sectional and longitudinal age-specific associations of *FTO* rs9939609 with BMI and the risk of obesity were examined in Chinese children and adolescents. Consistent with the findings from the Dutch Children Cohort study [12], we found that rs9939609 was positively associated with BMI and obesity in adolescents starting at ages 12–14 years or during puberty, particularly among girls. It is here postulated that the function of *FTO* in individuals of Chinese ancestry may be related to changes in DNA methylation at puberty. However, the age of onset of the association was later than that among European population, which was reported to be 7 years of age or earlier [7]. This difference may be explained by the diversity in genetic basis and phenotype of obesity in European and non-European populations.

The present findings suggested that the association of rs9939609 with obesity-related traits in Chinese children and adolescents also changes with age. The AA genotype was associated with gains in BMI at the 6-year follow-up. It is possible that changes in gene expression over the developmental time can have a profound influence on phenotype [21]. One recent animal study showed that global germline loss of *Fto* had a dramatic effect on body composition and resulted in stunted growth and some significant lethality, but loss during adulthood was better tolerated and reduced lean mass and increased fat mass [22]. For this reason, future work should focus on determining whether gene expression is in the form of temporal-specific pattern or whether the effects of expressed transcripts simply accumulate over time. Additionally, genetic influences may drive environmental exposure. Individuals at genetic risk for obesity may be more likely to select obesogenic environments correlated with their genetic propensities [6]. The accumulative effect of environmental risk factors may activate gene activity during the development of complex organisms. Previous findings have shown that both sedentary behavior and physical activity might modulate the effects of genetic variants on the risk of childhood obesity [17]. In mice, the expression of *FTO* in the arcuate nucleus (ARC) of the hypothalamus varied as a function of nutritional status (e.g., feeding and fasting) [23]. For mice in the fasted state, *FTO* mRNA levels in the ARC were reduced by approximately 60%. The findings of animal studies provide evidence to support gene-environment interplay in the association between *FTO* and obesity.

The current study also showed that the associations of the rs9939609 A allele in *FTO* with BMI and obesity were stronger in girls than in boys, and the associations persisted into late puberty in girls but not in boys. These findings were consistent with a previous study in Swedish children and adolescents [9]. However, no significant difference in *FTO* associations was found between boys and girls in a combined sample of non-Hispanic white and African American children and adolescents in two longitudinal studies [8]. Other studies have found rs9939609 to be associated with BMI in both sexes [4], [24]. These conflicting results may be due to low statistical power resulting from small sample sizes in subgroups stratified by sex, the low minor allele frequency (MAF) in children of Chinese ancestry, and the minor effect of the variant. The sex difference could also be explained by the different patterns of fat mass deposition and hormone levels between males and females. Population-based studies with large sample sizes will be crucial for further exploration of possible interactions with sex.

This study was based on a large cross-sectional population with different ages and a longitudinal sub-cohort with a follow-up visit 6 years later, allowing assessment of BMI and obesity in childhood (6–11 years) and adolescence (12–18 years). However, some limitations should be noticed. Although the statistical power is sufficient to detect the overall association of rs9939609 with obesity, it was not enough to detect age-specific or sex-specific associations between the variants and obesity-related traits. In addition, only one SNP of *FTO* was analyzed in the present study. Data collection on lifestyle (e.g., eating habit and physical activity) was not available at the 6-year follow-up. These variables were adjusted for as covariates using baseline level only. Thus, it is unclear whether the identified association is due to this specific sequence variant, gene-gene interaction, or gene-environment interaction.

In conclusion, the present findings suggest that the association between common variant rs9939609 in the *FTO* gene and obesity-related traits becomes evident after 12–14 years of age. It persists into late puberty only in girls. Results show that the positive association of *FTO* rs9939609 with BMI and the risk of obesity may change from childhood into adolescence in this Chinese population. Further longitudinal population-based studies with adequate statistical power are warranted. These must involve careful collection of information over the course of many years. Future mechanistic studies on the aetiology of obesity should span different age groups. If the age- and sex-specific genetic basis of the development of obesity could be confirmed, it could help guide in the prevention and management of obesity, especially during adolescence.

## Supporting Information

Table S1 Association of *FTO* rs9939609 with waist circumference separated by sex and age group.
(DOC)

Table S2 Association of *FTO* rs9939609 with waist-to-height ratio separated by sex and age group.
(DOC)

Table S3 Association of *FTO* rs9939609 with fat mass percentage separated by sex and age group.
(DOC)

## References

[1] Lobstein T, Baur L, Uauy R; IASO International Obesity Task Force (2004) Obesity in children and young people: a crisis in public health. Obes Rev (Suppl 1): 4–104. 10.1111/j.1467-789X.2004.00133.x 15096099

[2] Wardle J, Carnell S, Haworth CM, Plomin R (2008) Evidence for a strong genetic influence on childhood adiposity despite the force of the obesogenic environment. Am J Clin Nutr 87: 398–404. 18258631 10.1093/ajcn/87.2.398

[3] Elks CE, den Hoed M, Zhao JH, Sharp SJ, Wareham NJ, et al (2012) Variability in the heritability of body mass index: a systematic review and meta-regression. Front Endocrinol (Lausanne) 3: 29. 22645519 10.3389/fendo.2012.00029 PMC3355836

[4] Frayling TM, Timpson NJ, Weedon MN, Zeggini E, Freathy RM, et al (2007) A common variant in the FTO gene is associated with body mass index and predisposes to childhood and adult obesity. Science 316: 889–894. 17434869 10.1126/science.1141634 PMC2646098

[5] Loos RJ, Bouchard C (2008) FTO: the first gene contributing to common forms of human obesity. Obes Rev 9: 246–250. 18373508 10.1111/j.1467-789X.2008.00481.x

[6] Haworth CM, Carnell S, Meaburn EL, Davis OS, Plomin R, et al (2008) Increasing heritability of BMI and stronger associations with the FTO gene over childhood. Obesity (Silver Spring) 16: 2663–2668. 18846049 10.1038/oby.2008.434

[7] Hakanen M, Raitakari OT, Lehtimäki T, Peltonen N, Pahkala K, et al (2009) FTO genotype is associated with body mass index after the age of seven years but not with energy intake or leisure-time physical activity. J Clin Endocrinol Metab 94: 1281–1287. 19158205 10.1210/jc.2008-1199

[8] Hallman DM, Friedel VC, Eissa MA, Boerwinkle E, Huber JC Jr, et al (2012) The association of variants in the FTO gene with longitudinal body mass index profiles in non-Hispanic white children and adolescents. Int J Obes (Lond) 36: 61–68. 21986706 10.1038/ijo.2011.190 PMC3495000

[9] Jacobsson JA, Danielsson P, Svensson V, Klovins J, Gyllensten U, et al (2008) Major gender difference in association of FTO gene variant among severely obese children with obesity and obesity related phenotypes. Biochem Biophys Res Commun 368: 476–482. 18249188 10.1016/j.bbrc.2008.01.087

[10] Wang J, Mei H, Chen W, Jiang Y, Sun W, et al (2012) Study of eight GWAS-identified common variants for association with obesity-related indices in Chinese children at puberty. Int J Obes (Lond) 36: 542–547. 22083549 10.1038/ijo.2011.218

[11] López-Bermejo A, Petry CJ, Díaz M, Sebastiani G, de Zegher F, et al (2008) The association between the FTO gene and fat mass in humans develops by the postnatal age of two weeks. J Clin Endocrinol Metab 93: 1501–1505. 18252780 10.1210/jc.2007-2343

[12] Rutters F, Nieuwenhuizen AG, Bouwman F, Mariman E, Westerterp-Plantenga MS (2011) Associations between a single nucleotide polymorphism of the FTO Gene (rs9939609) and obesity-related characteristics over time during puberty in a Dutch children cohort. J Clin Endocrinol Metab 96: E939–E942. 21411553 10.1210/jc.2010-2413

[13] Xi B, Shen Y, Zhang M, Liu X, Zhao X, et al (2010) The common rs9939609 variant of the fat mass and obesity-associated gene is associated with obesity risk in children and adolescents of Beijing, China. BMC Med Genet 11: 107. 20598163 10.1186/1471-2350-11-107 PMC2914647

[14] Shan XY, Xi B, Cheng H, Hou DQ, Wang Y, Mi J (2010) Prevalence and behavioral risk factors of overweight and obesity among children aged 2–18 in Beijing, China. Int J Pediatr Obes 5: 383–389. 20233154 10.3109/17477160903572001

[15] Cole TJ, Bellizzi MC, Flegal KM, Dietz WH (2000) Establishing a standard definition for child overweight and obesity worldwide: international survey. BMJ 320: 1240–1243. 10797032 10.1136/bmj.320.7244.1240 PMC27365

[16] Marshall WA, Tanner JM (1986) Puberty. In: Falkner F, Tanner JM, eds. Human Growth: A Comprehensive Treatise, 2nd edu, Vol 2, New York and London: plenum press, 171–210p.

[17] Xi B, Wang C, Wu L, Zhang M, Shen Y, et al (2011) Influence of physical inactivity on associations between single nucleotide polymorphisms and genetic predisposition to childhood obesity. Am J Epidemiol 173: 1256–1262. 21527513 10.1093/aje/kwr008

[18] Germer S, Higuchi R (2003) Homogeneous allele-specific PCR in SNP genotyping. Methods Mol Biol 212: 197–214. 12491912 10.1385/1-59259-327-5:197

[19] Hardy R, Wills AK, Wong A, Elks CE, Wareham NJ, et al (2010) Life course variations in the associations between FTO and MC4R gene variants and body size. Hum Mol Genet 19: 545–552. 19880856 10.1093/hmg/ddp504 PMC2798720

[20] Graff M, Ngwa JS, Workalemahu T, Homuth G, Schipf S, et al (2013) Genome-wide analysis of BMI in adolescents and young adults reveals additional insight into the effects of genetic loci over the life course. Hum Mol Genet 22: 3597–3607. 23669352 10.1093/hmg/ddt205 PMC3736869

[21] Bergen SE, Gardner CO, Kendler KS (2007) Age-related changes in heritability of behavioral phenotypes over adolescence and young adulthood: a meta-analysis. Twin Res Hum Genet 10: 423–433. 17564500 10.1375/twin.10.3.423

[22] McMurray F, Church CD, Larder R, Nicholson G, Wells S, et al (2013) Adult Onset Global Loss of the Fto Gene Alters Body Composition and Metabolism in the Mouse. PLoS Genet 9: e1003166. 23300482 10.1371/journal.pgen.1003166 PMC3536712

[23] Gerken T, Girard CA, Tung YC, Webby CJ, Saudek V, et al (2007) The obesity-associated FTO Gene encodes a 2-oxoglutarate dependent nucleic acid demethylase. Science 318: 1469–1472. 17991826 10.1126/science.1151710 PMC2668859

[24] Qi L, Kang K, Zhang C, van Dam RM, Kraft P, et al (2008) Fat mass-and obesity-associated (FTO) gene variant is associated with obesity: longitudinal analyses in two cohort studies and functional test. Diabetes 57: 3145–3151. 18647953 10.2337/db08-0006 PMC2570413

