# Supplementary material for: Age- and Sex-Dependent Association between FTO rs9939609 and Obesity-Related Traits in Chinese Children and Adolescents
Source: PLoS One. 2014 May 14;9(5):e97545. doi: 10.1371/journal.pone.0097545 (PMC4020831; doi:10.1371/journal.pone.0097545)
Supplement: Table S1 — Association of FTO rs9939609 with waist circumference separated by sex and age group. (DOC) [file pone.0097545.s001.doc]

**Table S1.** Association of *FTO* rs9939609 with waist circumference separated by sex and age group

| **Sex** | **Age (years)** | **WC, cm, Mean (SD)** | | | ***P* value for trend** | **Change in WC per A allele ^a^** | |
| --- | --- | --- | --- | --- | --- | --- | --- |
|  |  | **TT** | **TA** | **AA** |  | ***β*** | **95%CI** |
| All | 6~8 | 18.6 (4.0) | 18.8 (4.3) | 19.9 (3.5) | 0.972 | 0.03 | -1.76, 1.83 |
|  | 9~11 | 21.0 (4.4) | 21.6 (4.3) | 21.6 (4.8) | 0.173 | 0.95 | -0.41, 2.31 |
|  | 12~14 | 22.3 (4.9) | 23.5 (4.6) | 24.7 (6.0) | **< 0.001** | **2.96** | **1.41, 4.52** |
|  | 15~18 | 23.6 (4.9) | 24.8 (4.6) | 25.3 (4.9) | **0.011** | **2.12** | **0.48, 3.75** |
|  | All | 71.7 (12.9) | 73.6 (12.9) | 75.4 (14.3) | **< 0.001** | **1.72** | **0.92, 2.52** |
|  |  |  |  |  |  |  |  |
| Boys | 6~8 | 64.0 (10.8) | 65.3 (11.5) | 67.3 (11.8) | 0.413 | 1.03 | -1.45, 3.51 |
|  | 9~11 | 73.3 (12.2) | 74.1 (12.1) | 77.0 (13.3) | 0.327 | 1.00 | -1.00, 3.00 |
|  | 12~14 | 78.2 (13.6) | 80.2 (12.3) | 98.8 (7.7) | **0.006** | **3.68** | **1.05, 6.31** |
|  | 15~18 | 83.5 (12.7) | 84.8 (12.0) | 80.4 (10.2) | 0.937 | 0.11 | -2.52, 2.73 |
|  | All | 75.4 (14.0) | 76.4 (13.6) | 80.2 (14.8) | **0.017** | **1.50** | **0.27, 2.72** |
|  |  |  |  |  |  |  |  |
| Girls | 6~8 | 59.5 (9.3) | 57.5 (8.6) | 60.8 (7.6) | 0.215 | -1.64 | -4.24, 0.96 |
|  | 9~11 | 67.0 (10.0) | 68.4 (10.2) | 64.6 (12.4) | 0.347 | 0.86 | -0.94, 2.67 |
|  | 12~14 | 70.6 (10.3) | 74.0 (10.6) | 71.0 (9.5) | **0.009** | **2.41** | **0.60, 4.22** |
|  | 15~18 | 71.3 (9.3) | 75.7 (9.7) | 78.2 (10.9) | **< 0.001** | **4.17** | **2.19, 6.14** |
|  | All | 68.1 (10.6) | 70.7 (11.5) | 69.7 (11.4) | **< 0.001** | **2.03** | **1.02, 3.04** |

Abbreviations: CI, confidence interval; *FTO*, fat mass- and obesity-associated gene; SD, standard deviation; WC, waist circumference.

^a^ Adjusted for sex and age.
