# Supplementary material for: Age- and Sex-Dependent Association between FTO rs9939609 and Obesity-Related Traits in Chinese Children and Adolescents
Source: PLoS One. 2014 May 14;9(5):e97545. doi: 10.1371/journal.pone.0097545 (PMC4020831; doi:10.1371/journal.pone.0097545)
Supplement: Table S2 — Association of FTO rs9939609 with waist-to-height ratio separated by sex and age group. (DOC) [file pone.0097545.s002.doc]

**Table S2.** Association of *FTO* rs9939609 with waist-to-height ratio separated by sex and age group

| **Sex** | **Age (years)** | **WHtR, Mean (SD)** | | | ***P* value for trend** | **Change in WHtR per A allele ^a^** | |
| --- | --- | --- | --- | --- | --- | --- | --- |
|  |  | **TT** | **TA** | **AA** |  | ***β*** | **95%CI** |
| All | 6~8 | 0.48 (0.06) | 0.48 (0.07) | 0.50 (0.07) | 0.749 | 0.002 | -0.010, 0.014 |
|  | 9~11 | 0.48 (0.07) | 0.49 (0.07) | 0.48 (0.08) | 0.351 | 0.004 | -0.004, 0.012 |
|  | 12~14 | 0.46 (0.07) | 0.48 (0.07) | 0.50 (0.10) | **< 0.001** | **0.019** | **0.009, 0.028** |
|  | 15~18 | 0.46 (0.07) | 0.48 (0.07) | 0.48 (0.06) | **0.008** | **0.013** | **0.004, 0.023** |
|  | All | 0.47 (0.07) | 0.48 (0.07) | 0.49 (0.08) | **< 0.001** | **0.01** | **0.006, 0.015** |
|  |  |  |  |  |  |  |  |
| Boys | 6~8 | 0.49 (0.07) | 0.51 (0.08) | 0.51 (0.08) | 0.167 | 0.011 | -0.005, 0.028 |
|  | 9~11 | 0.50 (0.07) | 0.50 (0.07) | 0.51 (0.08) | 0.619 | 0.003 | -0.009, 0.015 |
|  | 12~14 | 0.48 (0.08) | 0.49 (0.07) | 0.61 (0.06) | **0.002** | **0.024** | **0.009, 0.039** |
|  | 15~18 | 0.48 (0.07) | 0.49 (0.07) | 0.47 (0.05) | 0.843 | 0.002 | -0.014, 0.017 |
|  | All | 0.49 (0.07) | 0.5 (0.07) | 0.52 (0.08) | **0.009** | **0.01** | **0.002, 0.017** |
|  |  |  |  |  |  |  |  |
| Girls | 6~8 | 0.46 (0.06) | 0.45 (0.05) | 0.46 (0.03) | 0.1 | -0.014 | -0.030, 0.003 |
|  | 9~11 | 0.46 (0.06) | 0.47 (0.06) | 0.44 (0.08) | 0.403 | 0.005 | -0.006, 0.016 |
|  | 12~14 | 0.45 (0.06) | 0.47 (0.06) | 0.44 (0.06) | **0.009** | **0.015** | **0.004, 0.026** |
|  | 15~18 | 0.45 (0.06) | 0.47 (0.06) | 0.49 (0.07) | **< 0.001** | **0.025** | **0.013, 0.038** |
|  | All | 0.45 (0.06) | 0.47 (0.06) | 0.45 (0.07) | **0.001** | **0.011** | **0.005, 0.017** |

Abbreviations: CI, confidence interval; *FTO*, fat mass- and obesity-associated gene; SD, standard deviation; WHtR, waist-to-height ratio.

^a^ Adjusted for sex and age.
