# Supplementary material for: Age- and Sex-Dependent Association between FTO rs9939609 and Obesity-Related Traits in Chinese Children and Adolescents
Source: PLoS One. 2014 May 14;9(5):e97545. doi: 10.1371/journal.pone.0097545 (PMC4020831; doi:10.1371/journal.pone.0097545)
Supplement: Table S3 — Association of FTO rs9939609 with fat mass percentage separated by sex and age group. (DOC) [file pone.0097545.s003.doc]

**Table S3.** Association of *FTO* rs9939609 with fat mass percentage separated by sex and age group

| **Sex** | **Age (years)** | **FMP, %, Mean (SD)** | | | ***P* value for trend** | **Change in FMP per A allele ^a^** | |
| --- | --- | --- | --- | --- | --- | --- | --- |
|  |  | **TT** | **TA** | **AA** |  | ***β*** | **95%CI** |
| All | 6~8 | 20.2 (7.9) | 20.2 (8.2) | 20.8 (4.5) | 0.757 | -0.23 | -1.68, 1.22 |
|  | 9~11 | 23.8 (8.3) | 24.0 (7.7) | 22.8 (9.0) | 0.779 | 0.14 | -0.87, 1.16 |
|  | 12~14 | 24.3 (8.8) | 26.6 (8.5) | 28.9 (7.7) | **< 0.001** | **2.08** | **0.99, 3.16** |
|  | 15~18 | 26.6 (7.9) | 28.9 (8.3) | 28.1 (10.4) | **0.002** | **1.86** | **0.71, 3.00** |
|  | All | 24.1 (8.5) | 25.3 (8.6) | 25.7 (9.0) | **< 0.001** | **1.15** | **0.57, 1.73** |
|  |  |  |  |  |  |  |  |
| Boys | 6~8 | 21.0 (7.7) | 21.9 (8.3) | 21.4 (4.8) | 0.436 | 0.74 | -1.13, 2.62 |
|  | 9~11 | 24.1 (8.1) | 23.2 (7.7) | 25.0 (8.9) | 0.768 | -0.2 | -1.55, 1.15 |
|  | 12~14 | 21.7 (7.9) | 23.7 (7.5) | 32.8 (6.3) | **0.001** | **2.6** | **1.07, 4.12** |
|  | 15~18 | 24.8 (7.5) | 24.8 (6.4) | 23.5 (6.9) | 0.807 | -0.19 | -1.69, 1.32 |
|  | All | 23.0 (8.0) | 23.5 (7.5) | 25.5 (8.0) | 0.069 | 0.72 | -0.06, 1.49 |
|  |  |  |  |  |  |  |  |
| Girls | 6~8 | 19.4 (8.0) | 17.5 (7.5) | 19.4 (4.9) | 0.159 | -1.66 | -3.96, 0.65 |
|  | 9~11 | 23.4 (8.6) | 24.9 (7.7) | 19.7 (8.7) | 0.429 | 0.61 | -0.91, 2.14 |
|  | 12~14 | 26.7 (8.9) | 29.1 (8.6) | 27.0 (7.8) | **0.023** | **1.77** | **0.25, 3.29** |
|  | 15~18 | 28.3 (8.0) | 32.2 (8.3) | 36.3 (11.1) | **< 0.001** | **3.94** | **2.23, 5.64** |
|  | All | 25.1 (8.9) | 27.2 (9.2) | 26.0 (10.1) | **< 0.001** | **1.56** | **0.71, 2.40** |

Abbreviations: CI, confidence interval; FMP, fat mass percentage; *FTO*, fat mass- and obesity-associated gene; SD, standard deviation.

^a^ Adjusted for sex and age.
